# Diabetes-associated *Parvimonas* enrichment and altered lung microbiota profiles in lower respiratory tract infection: an analysis of 632 metagenomes

**DOI:** 10.1128/spectrum.04048-25

**Published:** 2026-07-17

**Authors:** Honghao Han, Qian Qian, Weiwei Wu, Jiangnan Yang, Ji Zhou, Wenkui Sun

**Affiliations:** 1 Department of Respiratory Medicine, Jiangsu Province Hospital/The First Affiliated Hospital with Nanjing Medical University 74734, Nanjing, Jiangsu, China; 2 Jiangsu Health Vocational College 205340 , Nanjing, Jiangsu, China; 3 Dinfectome Medical Technology Nanjing Co., Ltd 651565 , Nanjing, Jiangsu, China; Huazhong University of Science and Technology, Wuhan, China

**Keywords:** *Parvimonas*, diabetes, pneumonia, lung microbiota, metagenomic next-generation sequencing

## Abstract

The homeostasis of pulmonary microbiota is crucial in maintaining human health and modulating disease progression. The stability of pulmonary microbial flora may be associated with diabetes, yet the specific alterations remain poorly characterized. This retrospective observational study aims to analyze the profiles in pulmonary microbiota between individuals with and without diabetes, using metagenomic next-generation sequencing (mNGS). A total of 632 patients were sequentially enrolled, including 77 patients with both pneumonia and diabetes, 46 patients without either pneumonia or diabetes, 499 patients with pneumonia but without diabetes, and 10 diabetic patients without pneumonia. Pathogens in bronchoalveolar lavage fluid (BALF) specimens were detected using mNGS (DNA). The lung microbiota of diabetic individuals significantly differs from that of non-diabetic individuals in the non-lower respiratory tract infection (non-LRTI) cohort. *Parvimonas* was more abundant in the diabetic group. Compared to non-diabetic patients with LRTI, those with diabetes and LRTI showed an increased relative abundance of *Parvimonas,* but decreased relative abundances of *Prevotella* and *Malassezia*. Our analysis revealed a negative correlation between *Parvimonas* and *Malassezia*, alongside a positive association of *Parvimonas* with the expression of antimicrobial resistance genes ICR-Mc and RbpA. This suggests a potential association between *Parvimonas* enrichment and microbial dysbiosis during infection, although the underlying host–microbe interactions require further validation. Interestingly, *Parvimonas* abundance showed no significant association with HbA1c levels. Our findings suggest that *Parvimonas* enrichment is associated with diabetes-related alterations in lower respiratory tract microbiota. Whether microbiota-associated alterations represent clinically actionable targets in diabetic patients with pulmonary infections remains to be determined in prospective and interventional studies.

**IMPORTANCE:**

This study reveals significant differences in lung microbiota between diabetic and non-diabetic individuals. *Parvimonas* was enriched in the diabetic lung, and its abundance correlated with the expression of antimicrobial resistance genes, such as ICR-Mc and RbpA. Surprisingly, microbial dysbiosis was independent of HbA1c levels, indicating that mechanisms other than glycemic control contribute to infection progression. This study suggests that *Parvimonas* enrichment may be a diabetes-associated microbial feature in bronchoalveolar lavage fluid (BALF) microbiota, but its potential diagnostic or clinical relevance requires validation in future studies. Our work provides a scientific foundation for optimizing infection prevention and advancing precision anti-*Parvimonas* therapies.

## INTRODUCTION

Lung microbiota has gained significant attention in recent years as a crucial component of human microbiota (1). Traditionally, the lungs were considered to be a sterile environment. However, increasing evidence suggests that microbial flora exist in the lower respiratory tract of healthy individuals (2). Although the lung microbiota in healthy adults resembles that of the oropharynx, it also features a distinct microbial ecosystem (3). The lung microbiota is involved in the development and progression of respiratory diseases, including chronic obstructive pulmonary disease (COPD) (4), asthma (5), and lung cancer (6). The microbiota can interact with the host through inflammatory, immune, and metabolic pathways (7). Therefore, investigating changes in lung microbiota can provide valuable insights into the mechanisms of related diseases.

Patients with diabetes are at an increased risk of infections because of impaired immune function, particularly pulmonary infections (8). The incidence of pulmonary infections in diabetic patients is significantly higher than that in non-diabetic populations, with a concerning associated mortality rate (9, 10). However, the underlying mechanisms remain unclear. Furthermore, research characterizing alterations in the pulmonary microbiota of diabetic populations remains limited, particularly regarding their causal links to glycemic dysregulation. Research on diabetic mouse models reveals that diabetes can alter the composition of the pulmonary microbial community, and subsequently influence the host’s susceptibility to respiratory tract infections (11). Therefore, it is essential to investigate the changes in pulmonary microbiota in diabetic patients and to explore the mechanisms behind the increased susceptibility to infections.

Microbial culture, antigen or antibody immunology methods, and polymerase chain reaction have traditionally been used to identify pathogens responsible for lower respiratory tract infections (LRTI). However, these methods often encounter challenges, such as prolonged processing time and low detection rates (12). Metagenomic next-generation sequencing (mNGS), utilizing high-throughput sequencing and bioinformatics to detect multiple pathogens simultaneously, is a rapid and precise alternative (13). Compared to conventional diagnostic approaches, mNGS offers enhanced sensitivity and a broader detection range, and allows for the identification of a wider variety of microorganisms (14). Furthermore, mNGS provides crucial insights into microbial abundance and diversity (15) and thus is an invaluable tool for exploring microbial communities. In this study, mNGS was utilized to examine changes in the microbiota across different groups, including a diabetes mellitus (DM) group, an LRTI with DM group, an LRTI without DM group, and a control group. We aimed to characterize diabetes-associated alterations in the lung microbiota and to explore their potential associations with infection-related microbial and resistome changes.

## MATERIALS AND METHODS

### Study population and sample collection

This retrospective observational study enrolled 632 patients with suspected LRTI who visited Jiangsu Province Hospital between March 2021 and June 2023. The inclusion criteria were (i) age ≥18 years old, (ii) clinical suspicion of LRTI, (iii) submission of bronchoalveolar lavage fluid (BALF) for mNGS, and (iv) BALF samples processed following mNGS quality standards. Suspected LRTI was diagnosed on the basis of lung imaging showing new or progressive focal or diffuse infiltrates, along with at least one symptom of fever, cough, sputum production, shortness of breath, or dyspnea. LRTI was diagnosed using comprehensive reference standards, including microbiological tests and clinical assessments. This study was approved by the Institutional Review Committee of Jiangsu Province Hospital (Approval no. 2023-SR-139).

### Categorized patient groups

**Fig 1:**
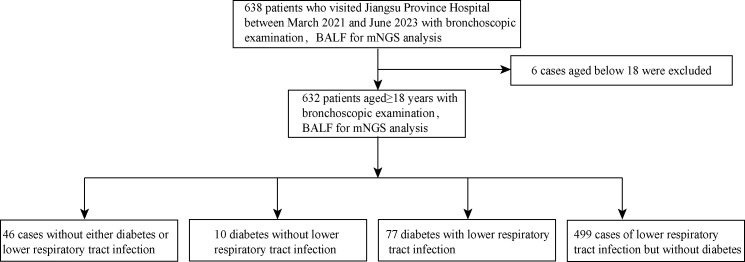
Flowchart of participants for the study.

From an initial cohort of 638 enrolled subjects, BALF was harvested for mNGS to detect pathogens. After exclusion of 6 patients aged below 18 years, the remaining 632 subjects were categorized into four groups: (i) 46 without either diabetes or lung infection, (ii) 10 with diabetes but without lung infection, (iii) 77 with both diabetes and lung infection, and (iv) 499 with lung infection but without diabetes. Among the 77 patients with diabetes complicated by lung infection, 63 underwent glycated hemoglobin (HbA1c) level testing after admission. Based on the test results, HbA1c values were distributed as follows: 14 patients ≤6.5%, 9 patients 6.51%–7.0%, 10 patients 7.01%–7.5%, 10 patients 7.51%–8.5%, 8 patients 8.51%–9.5%, and 12 patients >9.5%. They were further categorized into two subgroups: 33 patients with HbA1c levels less than or equal to 7.5%, and 30 patients with HbA1c levels above 7.5%. This selection process is detailed in Fig. 1.

### Specimen collection and processing

In this study, BALF specimens from patients with suspected pulmonary infection were collected by experienced clinicians following standardized procedures. Prior to the procedure, the nasal and oral cavities of each patient were thoroughly cleansed with saline to minimize external contamination. The patients were administered sedatives or local anesthetics as needed to ensure a comfortable and safe examination. All bronchial tubes were thoroughly examined under an electronic bronchoscope. Guided by the findings from chest CT scans, we brushed the identified lesions to ensure that the BALF collection accurately targeted the abnormal areas. For localized lesions, we selected the newly emerging or progressively infiltrative lesions. For diffuse lesions, we chose the right middle lobe or the lingula of the left upper lobe. The collection was carried out by infusing about 100 mL of sterile saline into the target bronchus at 37°C. The first 20 mL of fluid was discarded to prevent contamination, ensuring the purity and reliability of the collected samples. The recovered lavage solutions were promptly sent to the laboratory for analysis.

### Metagenomic next-generation sequencing

(i) Nucleic acid extraction: BALF samples were obtained following standardized protocols. DNA was extracted using a TIANamp Micro DNA kit (Tiangen Biotech, Beijing, China) by strictly adhering to the manufacturer’s guidelines. Each batch included a no-template control to ensure accuracy, and DNA was quantified, and quality was assessed using Qubit and NanoDrop devices (Thermo Fisher Scientific). (ii) Library preparation and sequencing: DNA libraries were prepared using the Hieff NGS C130P2 OnePot II DNA library prep kit for MGI (Yeasen Biotechnology) following the manufacturer’s protocols. The DNA libraries were then quality-checked using Agilent 2100. The libraries were sequenced with a 50 bp single-end approach on the MGISEQ-200 platform (MGI Tech, China). (iii) Bioinformatics analysis: high-quality sequencing data were selected after the exclusion of low-quality reads, adapter contamination, duplicated reads, and short reads (less than 36 bp) using fastq 0.23.2. Human host sequences were excluded by mapping the reads to the human reference genome (hs37d5) using Bowtie2 2.2.6. Host reads were removed computationally during bioinformatic processing, and only non-human reads were retained for downstream analyses. The remaining data were aligned with the NCBI microorganism genome database to identify the microorganisms in the samples. We used Kraken2 for the sequence alignment and identification of microorganisms. To ensure the accuracy and reliability of our results, we set the confidence threshold at 0.65. Additionally, to be included in the subsequent analysis, each species detected in the samples had to have a minimum of 5 reads. (iv) Microbial flora analysis: statistical analysis was performed on R software 4.3.1. Alpha diversity was estimated using the ACE index, Chao 1 index, Shannon index, or Simpson index based on the taxonomic profile of each sample. Beta diversity was assessed using the Bray-Curtis measure. In our study, to standardize the diversity indices for comparison between groups, we used the relative abundance of microbial species rather than raw read counts. Specifically, we converted the raw data for each species in a sample to relative abundance, which is the proportion of that species’ counts relative to the total counts in the sample. This approach allows for normalization without the need for data rarefaction, ensuring that we retain as much information as possible from the data set while still enabling valid comparisons between samples. These indices were compared between two groups using the Wilcoxon rank sum test. The results were then visualized on principal coordinate analysis (PCoA) plots. PERMANOVA was performed with the R package vegan (v 2.5.6) to analyze Bray-Curtis distance in different groups. Differential relative abundance of taxonomic groups at the species/genus level was tested by using the Kruskal-Wallis rank sum test (Kruskal.test in R package). The genera that exhibited a mean relative abundance of more than 0.1% and a detection rate over 10% across all samples were compared. DIAMOND was used to map reads against the CARD database to identify resistance genes. Resistance genes were then quantified and normalized using reads per kilobase per million (RPKM). RPKM was calculated as (C × 10^9^)/(L × N), where C is the number of reads mapped to a given resistance gene, L is the gene length in base pairs, and N is the total number of non-human high-quality reads retained in the sample after host-read removal.

### Statistical analysis

Continuous variables were compared between groups using the Mann-Whitney U test, whereas categorical variables were compared using the Chi-square test or Fisher’s exact test. Pearson correlation coefficient analysis was performed to assess the relationships between *Parvimonas* and *Prevotella*, *Malassezia*, as well as the antimicrobial resistance genes ICR-Mc and RbpA. A *P* value less than 0.05 was considered significant. Statistical analyses were performed using SPSS 27 (IBM Corporation).

## RESULTS

### Patient characteristics

A total of 632 patients were recruited, including 77 pneumonia patients with diabetes (LRTI with DM), 46 patients without either pneumonia or diabetes (non-DM), 10 diabetic patients without pneumonia (DM), and 499 patients with pneumonia but without diabetes (LRTI without DM). The clinical characteristics of the patients with or without diabetes were somewhat different (Tables 1 and 2 and Tables S1 to S3). Among non-LRTI patients, the diabetic group was significantly older at hospital admission and had a higher proportion of hypertension compared to the non-diabetic group.

**TABLE 1:** Demographic and baseline characteristics of patients^*b*^

	Non-LRTI group		
	DM (*n* = 10)	non-DM (*n* = 46)	*P* value^*a*^
Age, mean (range), years	60.8 (44–71)	51.0 (22–74)	0.031
Sex, female, *n* (%)	4 (40.0)	23 (50.0)	0.822
BMI	22.76 ± 3.59	23.40 ± 3.60	0.668
Any comorbidity, *n* (%)			
Hypertension	5 (50.0)	7 (15.2)	0.015
Cardiovascular disease	1 (10.0)	2 (4.3)	0.452
COPD	0	4 (8.6)	NA
Malignancy	1 (10.0)	7 (15.2)	1
Chronic liver disease	0	2 (4.3)	NA
Hematological disease	0	0	NA
Connective tissue disease	0	2 (4.3)	NA
Renal disease	0	0	NA
Hospital, mean (range), days	10.3 (2–40)	6.8 (3–14)	0.187

^*a*^ Diabetics without pneumonia vs patients without either pneumonia or diabetes group.

^*b*^ BMI, body mass index; COPD, chronic obstructive pulmonary disease; LRTI, lower respiratory tract infection; DM, diabetes mellitus; NA, not applicable.

**TABLE 2:** Laboratory findings of patients^*b*^

	LRTI group		
	LRTI with DM (*n* = 77)	LRTI without DM (*n* = 499)	*P* value^*a*^
White blood cell count, ×10^9^/L			
<4	6 (7.8)	6 (1.2)	<0.001
4–10	56 (72.7)	407 (82.7)	
>10	15 (19.5)	79 (16.1)	
Percentage of neutrophils			
<40	2 (26.0)	11 (2.2)	0.006
40–75	52 (67.5)	389 (79.1)	
>75	23 (29.9)	92 (18.7)	
Percentage of lymphocytes			
<20	41 (53.2)	160 (32.5)	0.002
20–50	35 (45.5)	326 (66.3)	
>50	1 (13.0)	6 (12.2)	
CRP, mg/L			
<10	22 (37.3)	209 (55.3)	0.021
10–50	13 (22.0)	86 (22.8)	
51–90	12 (20.3)	43 (11.4)	
>90	12 (20.3)	40 (10.6)	
Procalcitonin, ng/mL			
<0.1	17 (31.5)	151 (49.3)	0.094
0.1–0.24	25 (46.3)	112 (36.6)	
0.25–0.5	5 (9.3)	16 (5.2)	
>0.5	7 (13.0)	27 (8.8)	

^*a*^ Pneumonia patients with diabetes group vs pneumonia patients without diabetes group.

^*b*^ LRTI, lower respiratory tract infection; DM, diabetes mellitus; CRP, C-reactive protein.

In the LRTI cohort, diabetic patients were significantly older, had higher BMI, a lower proportion of females, elevated inflammatory markers, and longer mean hospital stays than non-diabetics, with all differences being statistically significant. Within the diabetic LRTI subgroup, no statistically significant differences in age distribution, sex ratio, or comorbidities were observed between low and high glycated hemoglobin (HbA1c) groups.

### Elevated *Parvimonas* in diabetic lungs under non-infected conditions

**Fig 2:**
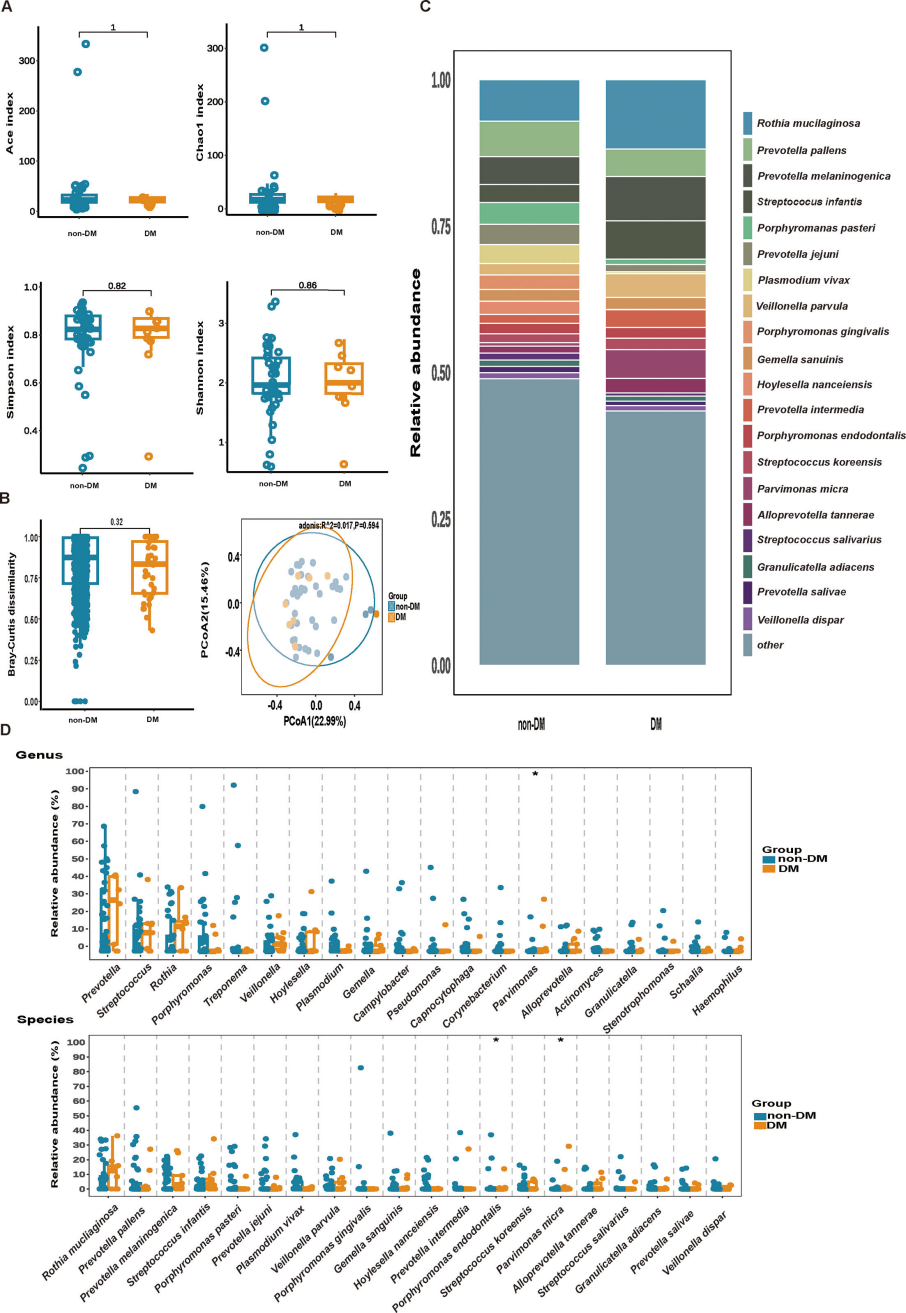
Comparison of pulmonary microbial communities between the DM group and the non-DM group. (**A**) Alpha diversity at the species level, including the ACE, Chao1, Shannon, and Simpson indices. The adjusted *P* value is shown at the top of the histogram. (**B**) PCoA1 and Bray-Curtis distance graphs to describe beta diversity. Each point represents a sample from each group. (**C**) Bar chart of the top 20 species with relative abundance in the two groups. Each color bar represents a type of bacteria. (**D**) Comparison of differences in relative abundance of microbial communities between the two groups at both species and genus levels. *P < 0.05.

To investigate the association of diabetes with the lung microbiota, we compared the lung microbial communities between the DM group and the non-DM group. In alpha diversity indices, no significant differences between the two groups were found at the species level in ACE, Chao1, Shannon, or Simpson index (Fig. 2A). Microbiota β-diversity analysis based on Bray–Curtis distance showed no significant difference between the DM and non-DM groups at the species level, as assessed by PERMANOVA (R² = 0.017, *P* = 0.594; Fig. 2B). We further analyzed the relative abundance of the top 20 species in each group at the species level (Fig. 2C). *Parvimonas micra* (*P* = 0.047) and *Porphyromonas endodontalis* (*P* = 0.049) showed higher relative abundance in diabetic individuals compared with non-diabetic individuals (Fig. 2D). We also explored the broader taxonomic context by examining relative abundance at the genus level. Similarly, *Parvimonas* was significantly more abundant in diabetic individuals at the genus level (*P* = 0.047). The stability of lung microbiota was influenced by diabetes concerning specific microbial compositions.

### Diabetes-associated lung microbiota alterations during lower respiratory tract infection

**Fig 3:**
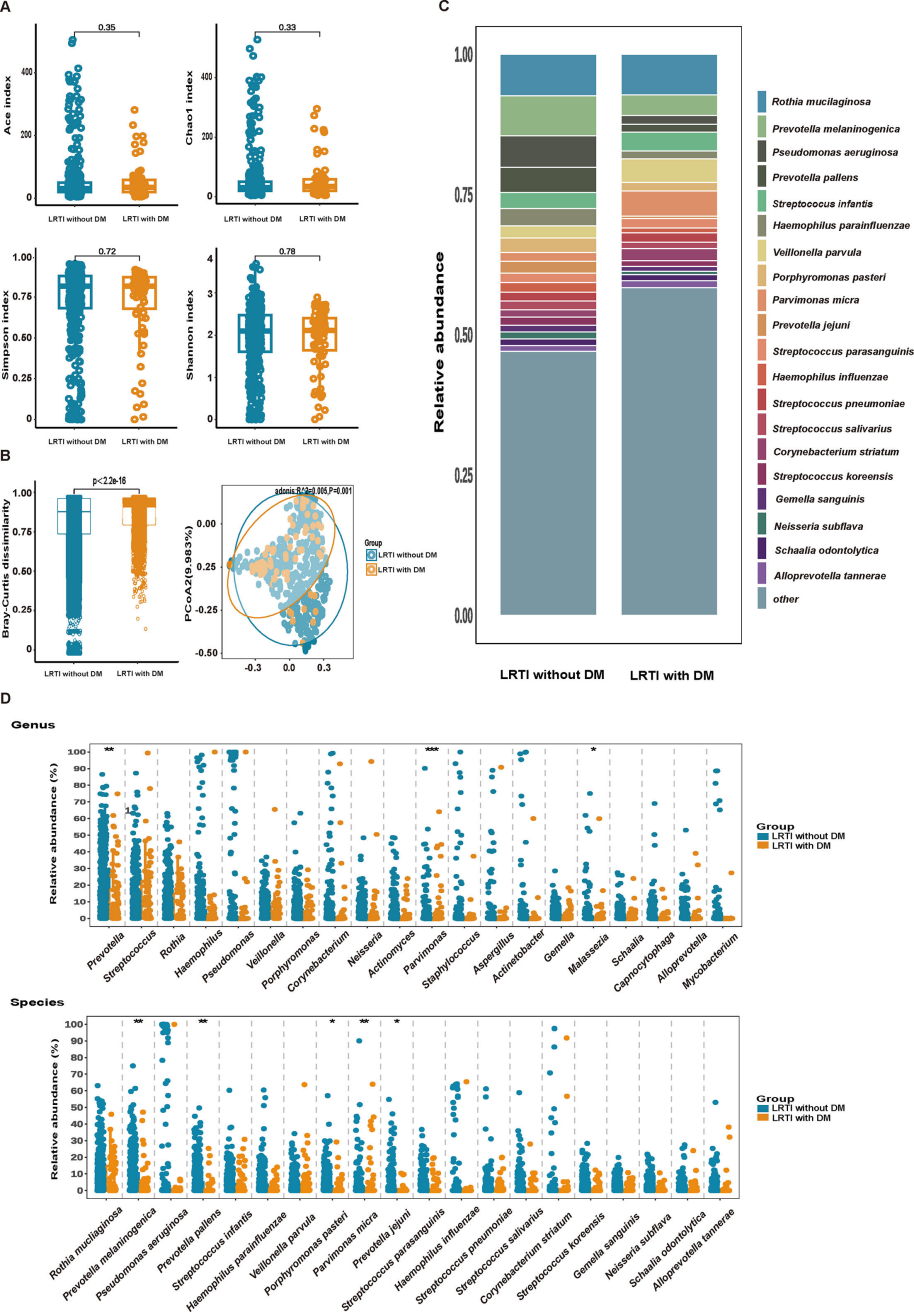
Comparison of pulmonary microbial flora between the LRTI with DM group and the LRTI without DM group. (**A**) Alpha diversity of ACE, Chao1, Shannon, and Simpson indices. The adjusted *P* value is listed at the top of the bar chart. (**B**) PCoA1 and Bray-Curtis distance graphs to describe beta diversity. Each point represents a sample from each group. (**C**) Bar chart of the top 20 species with relative abundance in the two groups. Each color bar represents a type of bacteria. (**D**) Comparison of differences in relative abundance of microbial communities between the two groups at both species and genus levels. *P < 0.05, **P < 0.01, ***P < 0.001.

To examine alterations in the pulmonary microbiota of LRTI patients with DM, we compared the microbiota between the LRTI without DM group and the LRTI with DM group. In alpha diversity indices, no significant differences between the two groups were found at the species level in ACE, Chao1, Shannon, or Simpson index (Fig. 3A).

Bray–Curtis distance-based β-diversity analysis showed a statistically significant but small difference in microbial community composition between the LRTI with DM and LRTI without DM groups, as assessed by PERMANOVA (R² = 0.005, *P* = 0.001; Fig. 3B). We further analyzed the relative abundance of the top 20 species in each group at the species level (Fig. 3C). Compared with non-diabetic patients with LRTI, those with diabetes and LRTI showed an increased relative abundance of *Parvimonas* (*P* = 0.001) but decreased relative abundances of *Prevotella* (*P* = 0.006) and *Malassezia* (*P* = 0.015) at the genus level (Fig. 3D). At the species level, the LRTI with DM group showed decreased relative abundances of *Prevotella melaninogenica* (*P* = 0.003), *Prevotella pallens* (*P* = 0.002), *Porphyromonas pasteri* (*P* = 0.032), and *Prevotella jejuni* (*P* = 0.042), but increased relative abundance of *Parvimonas micra* (*P* = 0.002) (Fig. 3D). These findings indicate significant changes in the diversity of lung microbiota in patients with LRTI and DM. Our results reveal that in the context of LRTI, the lung microbiota of diabetic patients exhibits significantly exacerbated dysbiosis compared to non-diabetic individuals, characterized by altered microbial diversity and compositional shifts.

### *Parvimonas* enrichment is associated with altered microbiota and resistome features in diabetic patients with LRTI

To investigate whether *Parvimonas* exacerbates microbiota dysbiosis under infectious conditions, we employed Pearson correlation coefficient analysis to assess the associations between *Parvimonas* and *Prevotella*, *Malassezia*, as well as the antimicrobial resistance genes ICR-Mc and RbpA (Table 3). The results revealed that in the LRTI with DM group, *Parvimonas* exhibited a statistically significant negative correlation with *Malassezia*, and a positive correlation with the upregulation of ICR-Mc and RbpA. In contrast, no significant associations were observed between *Parvimonas* and *Malassezia* or the antimicrobial resistance genes ICR-Mc and RbpA in the LRTI without DM group.

**TABLE 3:** Correlation of *Parvimonas* with differentially abundant bacterial genera and antimicrobial resistance genes in LRTI

Indicators (relative abundance)	Parvimonas in LRTI with DM		Parvimonas in LRTI without DM	
	*r*	*p*	*r*	*p*
Prevotella	0.066	0.573	0.032	0.479
Malassezia	−0.263	0.022	0.037	0.446
ICR-Mc	0.256	0.038	−0.021	0.672
RbpA	0.248	0.045	−0.041	0.395

### *Parvimonas* dysbiosis shows no significant association with HbA1c levels in the LRTI with DM group

**Fig 4:**
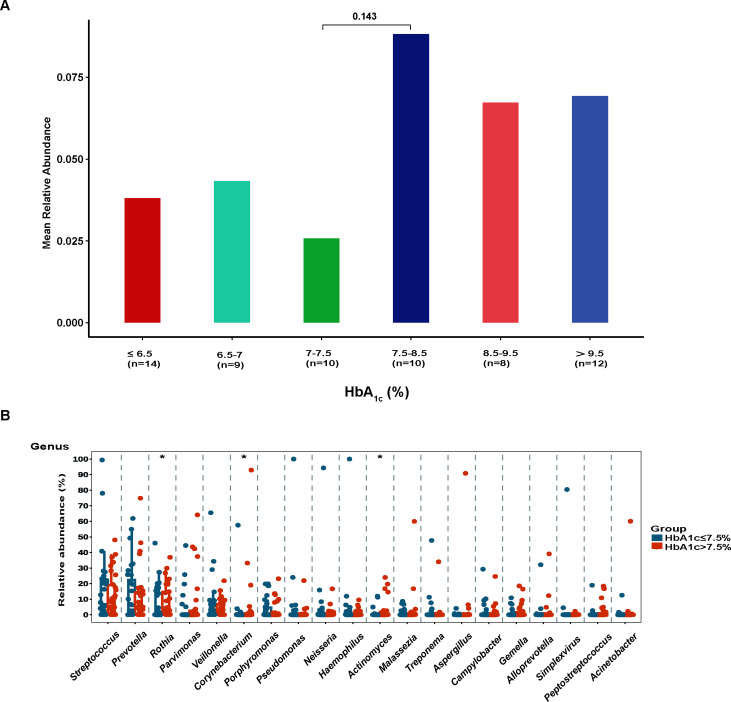
*Parvimonas* dysbiosis shows no significant association with HbA1c levels in the LRTI with DM group. (**A**) Patients were stratified into six subgroups based on HbA1c levels. No significant differences in *Parvimonas* abundance were observed across the subgroups. (**B**) Comparison of differences in relative abundance of microbial communities between groups (HbA1c ≤7.5% vs >7.5%) at genus levels. *P < 0.05.

To further explore the relationship between *Parvimonas* and HbA1c levels, patients were stratified into six subgroups based on HbA1c levels: 14 patients with HbA1c ≤6.5%, 9 patients with HbA1c 6.51%–7.0%, 10 patients with HbA1c 7.01%–7.5%, 10 patients with HbA1c 7.51%–8.5%, 8 patients with HbA1c 8.51%–9.5%, and 12 patients with HbA1c >9.5%. No significant differences in *Parvimonas* abundance were observed across the subgroups (Fig. 4A). A further binary classification was conducted by dividing patients into 33 individuals with HbA1c ≤7.5% and 30 individuals with HbA1c >7.5%, yet no significant differences in *Parvimonas* abundance were detected between the two groups (Fig. 4B). At the genus level, the relative abundances of *Rothia* (*P* = 0.049), *Corynebacterium* (*P* = 0.033), and *Actinomyces* (*P* = 0.018) were significantly increased in the high HbA1c group (Fig. 4B). These findings suggest that HbA1c-defined glycemic control alone may not fully explain *Parvimonas*-related microbial variation in this cohort, and further studies are needed to determine whether microbiota-associated alterations have clinical relevance.

## DISCUSSION

Our study provides novel insights into the relationship between diabetes and lung microbiota stability, employing mNGS. Our findings indicate that diabetes is associated with altered lung microbiota composition, characterized by enrichment of *Parvimonas*. In the context of diabetic lung infection, *Parvimonas* enrichment was accompanied by a reduction in potentially commensal taxa, such as *Malassezia* and co-occurred with an altered resistome profile, including increased abundance of antimicrobial resistance genes, such as ICR-Mc and RbpA. Notably, this series of microbial alterations showed no significant correlation with glycemic control as measured by HbA1c levels. This finding suggests that pulmonary microbiota alterations in diabetic patients may not be fully explained by HbA1c-defined glycemic control alone, and that other diabetes-related host factors may also be involved. This discovery offers new strategies for optimizing the prevention and management of lung infections in diabetic patients. In addition to glycemic control, future therapeutic strategies may prioritize targeted interventions against specific pathogens, such as *Parvimonas*, to restore microbial balance more effectively.

Our research reveals a distinct signature in the lung microbiota of diabetic individuals even in the absence of pulmonary infection (non-LRTI cohort), characterized by a significant enrichment of *Parvimonas*. Although the overall microbial diversity (α-diversity) and community structure dissimilarity (β-diversity) were not markedly altered in this non-LRTI cohort, the increased abundance of *Parvimonas* indicates a state of baseline dysbiosis. *Parvimonas* is an oral commensal bacterium that has potential pathogenicity in respiratory tract infections (16). The roles of *Parvimonas* in systemic diseases have garnered significant attention. *Parvimonas* is more abundant in the feces of colon cancer patients than in healthy individuals. This finding suggests a possible role for *Parvimonas* in promoting colorectal cancer occurrence and progression (17). Additionally, its close association with macrophage polarization may lead to immune suppression, increasing the risk of systemic infections in diabetic patients (18). Previous animal studies suggest that diabetes may alter pulmonary immune homeostasis and microbial community composition (11, 19). Nevertheless, whether these diabetes-related host changes contribute to the specific microbial pattern observed in our cohort, including *Parvimonas* enrichment and concomitant depletion of *Prevotella* and *Malassezia*, remains to be determined. Emerging evidence indicates that diabetes mellitus can induce dysbiosis of the intestinal microbiota. For instance, compared with healthy individuals, the abundance of *Bifidobacterium*, *Clostridia*, and *Firmicutes* in the intestinal microbiota of diabetic patients is significantly decreased (11). Additionally, lung microbiota is associated with various pulmonary diseases, including COPD and asthma (20). However, the influence of diabetes on the baseline composition of lung microbiota remains poorly characterized. Our study addresses this knowledge gap.

Building on the baseline dysbiosis observed in non-LRTI diabetics, our study reveals a more pronounced and complex disruption in the lung microbiota of diabetic individuals with LRTI. Specifically, compared to non-diabetic LRTI patients, diabetic LRTI patients exhibited a further increased relative abundance of *Parvimonas*, coupled with a significant decrease in the relative abundances of *Prevotella* and *Malassezia*. Critically, our correlation analysis revealed two distinct associations that characterize the altered microbial ecology in the diabetic lung. First, among all antimicrobial resistance genes quantified and normalized as RPKM through alignment against the CARD database, ICR-Mc and RbpA showed positive correlations with *Parvimonas* abundance in the LRTI with DM group in our exploratory correlation analysis, whereas no such correlations were observed in the LRTI without DM group. ICR-Mc is the protein that represents the closest known ortholog to the colistin resistance MCR-1 and MCR-2 PEtN transferases. The biofilm regulated by RbpA can protect bacteria from antibiotic and host immune attacks (21). Although ICR-Mc and RbpA have not been directly characterized in *Parvimonas*, their co-occurrence with *Parvimonas* in the diabetic lung may reflect shared niche adaptation within a disrupted polymicrobial ecosystem rather than direct genetic linkage or horizontal gene transfer. In polymicrobial environments, community-level antimicrobial tolerance or resistance can emerge through biofilm-mediated protection and metabolic interactions (22). Moreover, taxon-AMR gene co-occurrence in metagenomic data may indicate ecological co-selection or shared environmental selection pressures rather than direct carriage of specific resistance loci by a given taxon (23). These observations suggest that *Parvimonas* enrichment in the diabetic lung co-occurs with an altered resistome landscape, which may collectively pose additional challenges for infection management. Second, the negative correlation observed between *Parvimonas* and *Malassezia* suggests potential niche competition or antagonism. The concurrent depletion of *Prevotella* and *Malassezia*, together with the enrichment of *Parvimonas*, suggests an ecologically interconnected dysbiosis rather than isolated compositional shifts. In our data, *Prevotella* showed a negative correlation with *Parvimonas*, supporting the possibility of ecological antagonism or shared niche remodeling. Although direct causality cannot be inferred from the present metagenomic analysis, *Parvimonas* enrichment may reflect ecological release following the depletion of commensal taxa, or alternatively may contribute to a local microenvironment less favorable to *Prevotella* and *Malassezia* through metabolic competition, inflammatory modulation, or altered niche structure. The simultaneous reduction of bacterial and fungal commensal components may therefore indicate a weakened microbial network involved in colonization resistance and mucosal immune homeostasis. This pattern of “pathogen expansion, protector depletion, and resistance acquisition” paints a compelling picture of the critical pulmonary microenvironment in diabetic infection. *Parvimonas* enrichment in diabetic patients was accompanied by a reduction in commensal taxa, such as *Malassezia. Malassezia* is a lipophilic yeast genus typically associated with skin and mucosal surfaces (24). While *Malassezia* is often linked to skin conditions, its presence in the lung is documented, and its role remains debated (25). Several studies suggest that *Malassezia* can inhibit the proliferation of potential pathogens in the lung environment (26, 27). The concurrent reduction of *Prevotella* further compounds this ecological disruption. Although the role of *Prevotella* is species- and context-dependent, gut microbiome studies have linked certain *Prevotella* species to improved glucose tolerance and reduced visceral fat (28). Other studies suggest that some *Prevotella* species may confer protection in sepsis models (29, 30). In the respiratory tract, some *Prevotella* species are common commensals and may contribute to immune modulation and mucosal barrier maintenance (31). Therefore, the depletion of *Prevotella*, together with the reduction of *Malassezia*, may represent a loss of phylogenetically distinct but potentially homeostasis-associated microbial components within the lung ecosystem. In summary, this study reveals a distinct pulmonary dysbiosis pattern observed in diabetic patients with lower respiratory tract infection, marked by enrichment of *Parvimonas*, concomitant depletion of *Prevotella* and *Malassezia*, and an altered resistome landscape. Whether *Parvimonas* functionally contributes to resistome remodeling and microbial dysbiosis, or merely represents a bystander within a diabetes-associated dysbiotic ecosystem, remains to be determined through future mechanistic studies. To the best of our knowledge, this is the first report of such a diabetes-associated microbial co-occurrence pattern at the metagenomic level, offering a potential entry point for mechanistic investigation.

The glycosylated hemoglobin (HbA1c) level is closely associated with a patient’s immune function and risk of infection (32). Hyperglycemia not only impairs immune cell function but also creates an environment conducive to pathogen growth (33). Hyperglycemia can cause intestinal barrier dysfunction, elevating the risk of intestinal infections (34). We examined the alterations in the pulmonary microbiota of diabetic patients with pulmonary infections across different HbA1c levels. At the genus level, the relative abundance of *Rothia*, *Corynebacterium*, and *Actinomycetes* was significantly higher in the group with elevated HbA1c levels. *Rothia dentocariosa* is a conditionally pathogenic bacterium that may cause infective endocarditis in selected patients and give rise to a variety of clinical complications (35). In addition, the abundance of *Rothia dentocariosa* is significantly correlated with multiple cytokines, such as IL-1β, suggesting that it may be involved in the regulation of inflammation and immune responses (36). *Corynebacterium*, a genus of Gram-positive bacteria, is widely distributed on human skin and the upper respiratory tract. Most species within *Corynebacterium* are opportunistic pathogens, and typically do not cause disease but potentially become pathogenic when the host’s immune status is compromised (37). *Corynebacterium* was clearly identified as the responsible pathogen in a case analysis of 12 infectious diseases, and thus posed a potential threat to pulmonary health (38). *Actinomycetes* represent a group of Gram-positive bacteria exhibiting substantial biological activities and ecological functions (39). These microorganisms demonstrate associations with the phylum *Firmicutes* and may influence the equilibrium of the intestinal microbiota (40). These three types of bacteria are widely distributed across various human microbial communities and are crucial in maintaining the balance of microbial ecosystems. Our findings support an association between impaired glycemic control and alterations in the composition of the lung microbiota, suggesting a potential link between poor blood sugar regulation and specific components of the pulmonary microbial community. However, the observed microbial dysbiosis, *Parvimonas*, exhibited no significant association with HbA1c levels. Consequently, the treatment of diabetic patients with pulmonary infections requires not only rigorous glycemic control but also targeted interventions to address concomitant microbial dysbiosis.

The composition of the lung microbiota is remarkably different from that of the gut microbiota. Notably, the lung microbiome is characterized by low biomass and is primarily influenced by dynamic processes of microbial immigration and clearance (41). The intestinal microbiota exhibits a relatively high number of sequences and is predominantly composed of *Proteobacteria*, followed by *Actinobacteria*, *Firmicutes*, and *Bacteroidetes* (1). Diabetic individuals exhibit higher susceptibility to respiratory viral infections, such as influenza and SARS-CoV-2 (42). Compared with healthy individuals, the abundance of *Bifidobacterium*, *Clostridia*, and *Firmicutes* in the intestinal microbiota of diabetic patients is significantly decreased (11). These bacterial communities are closely associated with impaired glucose tolerance. In our cohort, *Parvimonas* was enriched in the lower respiratory tract microbiota of diabetic patients, suggesting that diabetes-associated microbial alterations may also involve the lung. However, this retrospective study was not designed to establish diagnostic performance. Future prospective studies with prespecified models, ROC/AUC analyses, and independent validation cohorts are needed to determine whether *Parvimonas* has diagnostic or predictive value.

Several limitations should be acknowledged. First, BALF is a low-biomass specimen, and *Parvimonas* is a typical oral anaerobe. Therefore, upper-airway or reagent-derived contamination remains a concern. Although BALF samples were collected using standardized bronchoscopy procedures and no-template controls were included in each sequencing batch, matched oral samples, saline/bronchoscope blanks, and extraction blanks were not available in this retrospective study. Thus, minor oral, procedural, or reagent-derived contamination cannot be completely excluded. Future prospective studies incorporating paired oral and BALF sampling, procedural and extraction controls, and absolute microbial load measurements are needed to validate the pulmonary origin and ecological relevance of *Parvimonas*. Second, because this study was based on clinically indicated bronchoscopy, healthy community controls were unavailable. In addition, BALF collection from individuals with diabetes alone, without LRTI or another pulmonary indication, is ethically difficult to justify in routine clinical practice because bronchoscopy is an invasive procedure. These constraints limit our ability to distinguish background respiratory microbiome features from diabetes- or infection-associated microbial changes, particularly under non-infectious conditions. Third, although the overall sample size was relatively large, the number of diabetic patients without LRTI was limited, reducing statistical power and making subgroup-level observations more susceptible to inter-individual variability. Accordingly, findings from this subgroup should be regarded as exploratory and hypothesis-generating, and the current data set does not allow us to determine whether diabetes independently contributes to lower respiratory tract microbiota dysbiosis in the absence of active infection. Finally, several demographic and clinical variables differed between the DM and non-DM groups, including age, BMI, sex distribution, and comorbidities. These imbalances may confound the observed microbiota differences and influence the statistical findings. In addition, because microbiome and resistome analyses involve comparisons across multiple taxa and genes, the possibility of false-positive associations cannot be fully excluded. Owing to the retrospective design, subgroup imbalance, the limited number of diabetic patients without LRTI, and the high-dimensional, sparse, and zero-inflated nature of microbiome data, robust multivariable adjustment for all relevant confounders was limited in the current data set and could have resulted in unstable estimates or model overfitting. Accordingly, our findings should be interpreted as associative and hypothesis-generating rather than as evidence that diabetes independently drives lower respiratory tract microbiota dysbiosis. Larger prospective cohorts with balanced subgroup sizes, appropriate healthy and disease control groups, paired contamination controls, and prespecified multivariable adjustment strategies are required to validate these findings and clarify the independent contribution of diabetes to lower respiratory tract microbiota alterations.

Despite these limitations, our findings suggest that diabetes is associated with differences in pulmonary microbiota composition, including enrichment of *Parvimonas*. In this cohort, the *Parvimonas*-related microbial pattern observed during lung infection was not clearly explained by HbA1c alone, suggesting that additional diabetes-related host or clinical factors may contribute to these microbiota alterations. These observations should be considered hypothesis-generating and warrant validation in larger prospective studies, as well as a mechanistic investigation into the role of *Parvimonas* and related microbial networks in diabetic patients with lung infection.

## Data Availability

All sequence data, excluding the human genome, from this study have been uploaded to the National Center for Biotechnology Information (NCBI) Sequence Read Archives under the project accession number CRA021551.

## SUPPLEMENTAL MATERIAL

Table S1 spectrum.04048-25-s0001.doc Demographic and baseline characteristics of patients in LRTI group.

Table S2 spectrum.04048-25-s0002.docx Laboratory findings of patients in LRTI with DM group.

Table S3 spectrum.04048-25-s0003.docx Demographic and baseline characteristics of patients in LRTI with DM group.

ASM does not own the copyrights to Supplemental Material that may be linked to, or accessed through, an article. The authors have granted ASM a non-exclusive, world-wide license to publish the Supplemental Material files. Please contact the corresponding author directly for reuse.
