# Supplementary material for: Diabetes-associated Parvimonas enrichment and altered lung microbiota profiles in lower respiratory tract infection: an analysis of 632 metagenomes
Source: Microbiol Spectr. 2026 Jul 17;14(8):e04048-25. doi: 10.1128/spectrum.04048-25 (PMC13436127; doi:10.1128/spectrum.04048-25)
Supplement: Supplementary file 1 [file spectrum.04048-25-s0002.docx]

**Supplementary Table 2. Laboratory Findings of Patients**

| LRTI with DM Group | | | |
| --- | --- | --- | --- |
|  | DM, HbA1c ≤ 7.5%  (n=33) | DM, HbA1c > 7.5%  (n=30) | *P*# |
| White blood cell count, ×10^9^/L |  |  |  |
| < 4 | 3 (9.1) | 2 (6.7) | 0.58 |
| 4-10 | 25 (75.8) | 20 (66.7) |  |
| > 10 | 5 (15.2) | 8 (26.7) |  |
| Percentage of neutrophils |  |  |  |
| < 20% | 17 (51.5) | 17 (56.7) | 0.613 |
| 20-50% | 16 (48.5) | 12 (40.0) |  |
| > 50% | 0 | 1 (3.3) |  |
| CRP, mg/L |  |  |  |
| < 10mg/L | 10 (40.0) | 8 (33.3) | 0.977 |
| 10-50 mg/L | 5 (20.0) | 6 (25.0) |  |
| 51-90 mg/L | 5 (20.0) | 5 (20.8) |  |
| > 90 mg/L | 5 (20.0) | 5 (20.8) |  |
| Procalcitonin, ng/mL |  |  |  |
| < 0.1ng/ml | 8 (33.3) | 7 (31.8) | 0.534 |
| 0.1-0.24 ng/mL | 11 (45.8) | 9 (40.9) |  |
| 0.25-0.5 ng/mL | 3 (12.5) | 1 (4.55) |  |
| > 0.5 ng/mL | 2 (8.3) | 5 (22.7) |  |

Data are presented as n (%); LRTI, lower respiratory tract infection; DM, Diabetes Mellitus; CRP, C-reactive protein; # diabetes with lower respiratory tract infection and glycosylated hemoglobin ≤ 7.5% vs. diabetes with lower respiratory tract infection and glycosylated hemoglobin > 7.5% group.
