# Supplementary material for: Diabetes-associated Parvimonas enrichment and altered lung microbiota profiles in lower respiratory tract infection: an analysis of 632 metagenomes
Source: Microbiol Spectr. 2026 Jul 17;14(8):e04048-25. doi: 10.1128/spectrum.04048-25 (PMC13436127; doi:10.1128/spectrum.04048-25)
Supplement: Supplementary file 2 [file spectrum.04048-25-s0003.docx]

**Supplementary Table 3. Demographic and Baseline Characteristics of Patients**

| LRTI with DM Group | | | |
| --- | --- | --- | --- |
|  | DM, HbA1c ≤ 7.5% (n=33) | DM, HbA1c > 7.5% (n=30) | *P*# |
| Age, mean (range), years | 63.0 (29-84) | 60.5 (40-76） | 0.319 |
| Sex, female, n (%) | 13 (39.4) | 6 (20.0) | 0.094 |
| BMI | 23.33 ± 3.34 | 20.76 ± 3.03 | 0.881 |
| Any comorbidity, n (%) |  |  |  |
| Hypertension | 18 (54.5) | 12 (40.0) | 0.248 |
| Cardiovascular disease | 3 (9.1) | 2 (6.7) | 1.0 |
| COPD | 5 (15.2) | 2 (6.7) | 0.504 |
| Malignancy | 4 (12.1) | 2 (6.7) | 0.759 |
| Chronic liver disease | 4 (12.1) | 4 (13.3) | 1.0 |
| Hematological disease | 3 (9.1) | 1 (3.3) | 0.675 |
| Connective tissue disease | 1 (3.0) | 1 (3.3) | 1.0 |
| Renal disease | 1 (3.0) | 2 (6.7) | 0.933 |
| Hospital, mean (range), days | 8.0 (3-26) | 9.0 (3-28) | 0.599 |

BMI, Body Mass Index; COPD, Chronic obstructive pulmonary disease; LRTI, lower respiratory tract infection; DM, diabetes mellitus; # diabetes with lower respiratory tract infection and glycosylated hemoglobin ≤ 7.5% vs. diabetes with lower respiratory tract infection and glycosylated hemoglobin > 7.5% group.
