# Supplementary material for: Diabetes-associated Parvimonas enrichment and altered lung microbiota profiles in lower respiratory tract infection: an analysis of 632 metagenomes
Source: Microbiol Spectr. 2026 Jul 17;14(8):e04048-25. doi: 10.1128/spectrum.04048-25 (PMC13436127; doi:10.1128/spectrum.04048-25)
Supplement: Supplementary file 3 [file spectrum.04048-25-s0001.doc]

**Supplementary Table 1. Demographic and Baseline Characteristics of Patients**

| LRTI Group | | | |
| --- | --- | --- | --- |
|  | LRTI with DM (n=77) | LRTI without DM(n=499) | *P*& |
| Age, mean (range), years | 62.0 (29-84) | 56.0 (19-88） | < 0.001 |
| Sex, female, n (%) | 22 (28.6) | 242 (48.5) | 0.001 |
| BMI | 23.77 ± 2.91 | 20.13 ± 2.03 | 0.034 |
| Any comorbidity, n (%) |  |  |  |
| Hypertension | 35 (45.5) | 116 (23.2) | < 0.001 |
| Cardiovascular disease | 6 (7.8) | 39 (7.8) | 0.994 |
| COPD | 7 (9.1) | 52 (10.4) | 0.72 |
| Malignancy | 9 (11.7) | 55 (11.0) | 0.863 |
| Chronic liver disease | 7 (9.1) | 25 (5.0) | 0.235 |
| Hematological disease | 4 (5.2) | 14 (2.8) | 0.441 |
| Connective tissue disease | 2 (2.6) | 26 (5.2) | 0.479 |
| Renal disease | 3 (3.9) | 6 (1.2) | 0.214 |
| Hospital, mean (range), days | 8.4 (3-28) | 7.17 (1-52) | 0.049 |

BMI, Body Mass Index; COPD, Chronic obstructive pulmonary disease; LRTI, lower respiratory tract infection; DM, diabetes mellitus; & pneumonia patients with diabetes group vs. pneumonia patients without diabetes group.
